# Germ Cell Tumor with Bronchial Fistula

**DOI:** 10.1155/2020/7650206

**Published:** 2020-02-25

**Authors:** María Katherinne Flórez Leguía, Paula Camila Riveros Calvete, Paulina Ojeda, Jorge Alberto Carrillo Bayona

**Affiliations:** ^1^Radiology Department, Universidad de Antioquia, Medellín, Colombia; ^2^Medical School, Universidad del Rosario, Bogotá, Colombia; ^3^Pathology Department, Hospital Universitario Mayor, Mederi, Bogotá, Colombia; ^4^Radiology Department, Thoracic Imaging Division, Hospital Universitario Mayor – Méderi, Universidad del Rosario, Bogotá, Colombia

## Abstract

Germ cell tumors account for 15% of anterior mediastinum tumors. Fistulas are abnormal communications between two surfaces covered by the epithelium. A fistula can occur between the bronchial tree and the adjacent anatomical structures secondary to variable etiologies. The main clinical manifestations of bronchial fistulas include hemoptysis, purulent cough, and pneumonia, which might threaten the patient's life. Diagnosis can be established with computed tomography, which shows direct and indirect signs of a fistulous tract. We present the case of a 25-year-old patient, with an embryonic carcinoma of the mediastinum, who developed a fistula between the mediastinal mass and the bronchial tree after chemotherapy and thoracic radiotherapy. We carried out a review of the literature about the epidemiological aspects and the physiopathology and the relevant radiological findings of this pathology.

## 1. Introduction

Germ cell tumors correspond to 15% of anterior mediastinum tumors in adults; they occur mainly in the second and fourth decade of life and are more frequent in men [1].

Fistulas are abnormal communications between two surfaces covered by the epithelium. The bronchial tree can develop fistulas with adjacent anatomical structures. Cases of anterior mediastinum teratomas with fistulas of the bronchial tree have been reported. Symptoms described in those patients include cough, hemoptysis, and dyspnea [2–4]. No cases of fistulas have been described between an embryonic carcinoma and the bronchial tree so far.

## 2. Case Presentation

**Figure 1:**
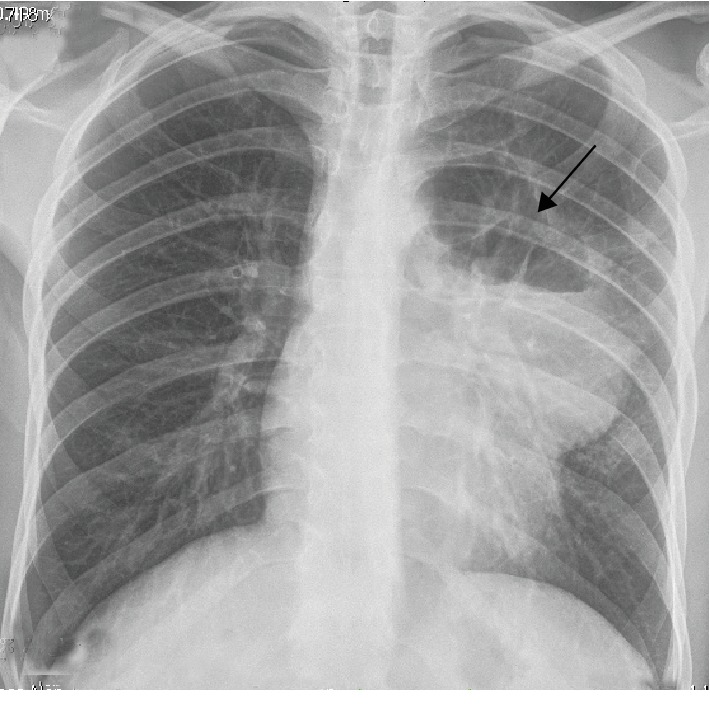
Male, 25-year-old patient with an anterior mediastinal mass (embryonal carcinoma germ cell tumor). Findings: cavitated mediastinal mass for the anterior segment of the left upper lobe (arrow). Technique: chest X-ray shows mediastinal mass.

**Figure 2:**
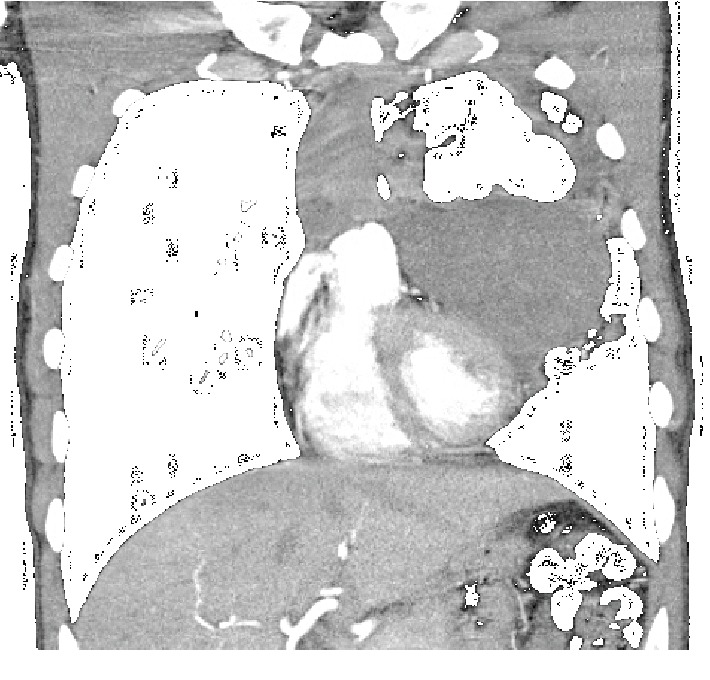
Male, 25-year-old patient with an anterior mediastinal mass (embryonal carcinoma germ cell tumor). Findings: cavitated mediastinal mass with soft tissue attenuation (arrow). Technique: contrasted computed tomography of the thorax, mediastinal window.

**Figure 3:**
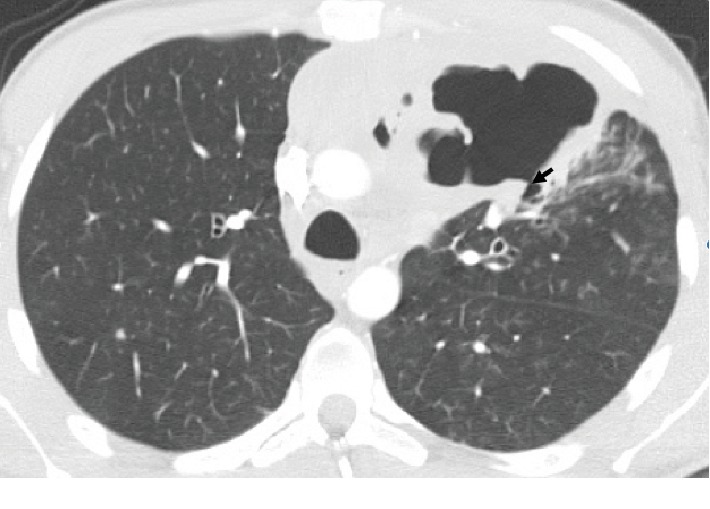
Male, 25-year-old patient with an anterior mediastinal mass (embryonal carcinoma germ cell tumor). Findings: a fistulous tract (arrow) between the cavitated mediastinal mass and the anterior segment of the left upper lobe bronchus is shown. Technique: contrasted computed tomography of the thorax, lung window. Chest CT with intravenous contrast, lung window.

A 25-year-old patient with an anterior mediastinal mass (embryonal carcinoma germ cell tumor) diagnosed by open surgery biopsy was treated with systemic chemotherapy (4 cycles, BEP protocol, with cisplatin, etoposide, and bleomycin) due to unresectability criteria. One month after the fourth cycle, he consulted for hemoptysis. The admission laboratory tests exhibited normochromic normocytic anemia (hemoglobin of 8.6). The chest X-ray (Figure 1) revealed a mediastinal mass, and the computed tomography (CT) showed a cavitated mass in the anterior mediastinum communicated with the bronchus for the anterior segment of the left upper lobe (Figures 2 and 3). Selective embolization was attempted, and it was unable to demonstrate active bleeding from bronchial arteries. The hemoptysis resolved spontaneously, and the patient was discharged from the hospital. Two weeks later, the patient developed a pulmonary infection and died.

## 3. Discussion

### 3.1. Etiology and Demographics

Nonseminomatous germ cell tumors are infrequent and have an estimated incidence of 16%. Embryonal carcinoma has an incidence of 3.2% of all mediastinal germ cell tumors [5].

Fistulas are abnormal communications between two surfaces covered by the epithelium. The fistula creation would be related to the rapid growth of these tumors with a mismatch between demand and blood supply. This mechanism leads to tumor necrosis allowing communication with adjacent structures. Finally, superinfection of the tumor may be associated with fistulas due to the inflammation that leads to tissue friability [6].

We describe the case of a fistula between an embryonic carcinoma in the anterior mediastinum with the bronchial tree, which appears after the fourth cycle of chemotherapy treatment (bleomycin, etoposide, and cisplatin). It is believed that the fistula developed secondary to chemotherapy because of the induction of tumor necrosis.

### 3.2. Clinical and Imaging Findings

Clinically, patients present chest pain (which usually is a sign of invasive spread of malignant neoplasia), cough, and dyspnea that can be explained by the compression or invasion of contiguous structures. Other less common symptoms are fever, hemoptysis, vena cava obstruction, dysphagia, Horner's syndrome, arrhythmias, and weight loss [1].

Radiologically, nonseminomatous germ cell tumors are manifested as anterior-lobulated mediastinal masses, with irregular or well circumscribed margins, that are associated with pleural and pericardial effusion and may also have local invasion. Chest CT usually exhibits a large mass of heterogeneous attenuation with areas of hemorrhage and necrosis, in up to 50% of the tumor [7]. The fat planes are usually obliterated, and the interface between the tumor and the lung is irregular.

**Figure 4:**
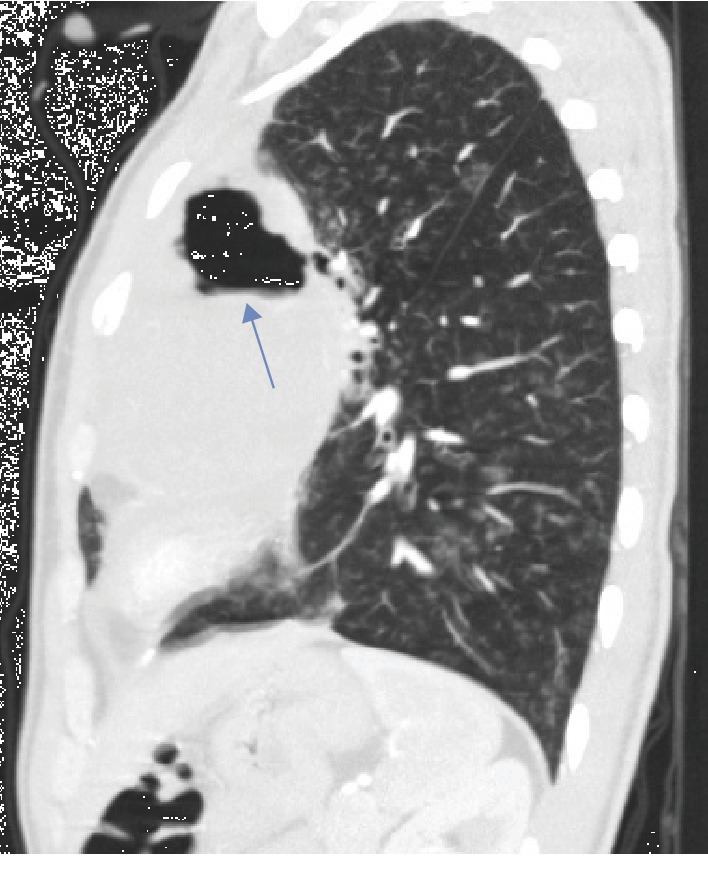
Male, 25-year-old patient with an anterior mediastinal mass (embryonal carcinoma germ cell tumor). Findings: cavitated mediastinal mass (arrow) and small centrilobular opacities scattered throughout the lung in a nonuniform distribution (arrowhead). Technique: contrasted computed tomography of the thorax, sagittal view.

CT is the primary imaging modality for suspected cases of bronchial tree fistulas; it demonstrates the fistulous tract and the possible cause (abscess, pneumonia, and tumor). It also gives information of the anatomical relationships, vascular structures (especially important in the context of hemoptysis), and the relationship with the diaphragm and mediastinum. Indirect signs of bronchial tree fistulas such as atelectasis, pneumonitis, and consolidation can be observed (Figure 4) [8]. In the present case, the diagnosis of bronchial fistula with embryonic carcinoma was made using chest CT that revealed the abnormal communication between the bronchus for the anterior segment of the left upper lobe and the mediastinal mass (Figures 2 and 3).

### 3.3. Treatment and Prognosis

The 5-year overall survival rate in patients with germ cell tumor treated with induction chemotherapy followed by surgery reported to range from 45.0% to 66.7% [9].

The fistulas require treatment because they are associated with hemoptysis, pneumonia, and acute respiratory distress syndrome compromising the patient's life [8]. The placement of a bronchial stent at the perforation site reduces the risk of complications [10].

### 3.4. Differential Diagnosis

The bronchial tree can develop fistulas with adjacent anatomical structures. A bronchopleural fistula is a communication between the pleura and bronchial tree, this type is the most common. The principal etiology of a bronchopleural fistula is a postoperative complication of a pulmonary resection [11]. Less frequent types of fistula include tracheoesophageal and gastrobronchial and fistulas of the bronchial tree with the bile duct or pancreas with bronchial tree.

## 4. Conclusion

The clinical picture of hemoptysis in a patient with a mediastinal mass should raise suspicion of a fistula of the mass with the bronchial tree. Chest CT allows diagnosis when abnormal communication or indirect signs of this pathology are demonstrated.
